# Metal chelation attenuates oxidative stress, inflammation, and vertical burn progression in a porcine brass comb burn model

**DOI:** 10.1016/j.redox.2021.102034

**Published:** 2021-06-08

**Authors:** Amina El Ayadi, John R. Salsbury, Perenlei Enkhbaatar, David N. Herndon, Naseem H. Ansari

**Affiliations:** a Department of Biochemistry & Molecular Biology, University of Texas Medical Branch, Galveston, TX, 77555-0647, USA; b Department of Surgery, University of Texas Medical Branch, Galveston, TX, 77555-0647, USA; c Department of Anesthesiology, University of Texas Medical Branch, Galveston, TX, 77555-0647, USA

**Keywords:** Thermal injury, Burn conversion, Iron chelation, Oxidative damage, Epithelial and endothelial cell death, Wound healing

## Abstract

Oxidative stress and inflammation may mediate cellular damage and tissue destruction as the burn wound continues to progress after the abatement of the initial insult. Since iron and calcium ions play key roles in oxidative stress, this study tested whether topical application of a metal chelator proprietary lotion (Livionex Formulation (LF) lotion), that contains disodium EDTA as a metal chelator and methyl sulfonyl methane (MSM) as a permeability enhancer, would prevent progression or reduce burn wound severity in a porcine model.

We have reported earlier that in a rat burn model, LF lotion reduces thermal injury progression. Here, we used the porcine brass comb burn model that closely mimics the human condition for contact burns and applied LF lotion every 8 h starting 15 min after the injury. We found that LF lotion reduces the depth of cell death as assessed by TUNEL staining and blood vessel blockage in the treated burn sites and interspaces. The protein expression of pro-inflammatory markers IL-6, TNF-a, and TNFα Converting Enzyme (TACE), and lipid aldehyde production (protein-HNE) was reduced with LF treatment. LF lotion reversed the burn-induced decrease in the aldehyde dehydrogenase (ALDH-1) expression in the burn sites and interspaces. These data show that a topically applied EDTA-containing lotion protects both vertical and horizontal burn progression when applied after thermal injury. Curbing burn wound conversion and halting the progression of second partial burn to third-degree full-thickness burn remains challenging when it comes to burn treatment strategies during the acute phase. Burn wound conversion can be reduced with targeted treatments to attenuate the oxidative and inflammatory response in the immediate aftermath of the injury. Our studies suggest that LF lotion could be such a targeted treatment.

## Highlights

• Burn progression manifest by increased expression of inflammatory cytokines and lipid aldehydes.
• Metal chelation reduces vertical progression of endothelial and mesenchymal cell death.
• Metal chelator containing lotion decreases the protein expression of IL-6, TNF-a, and TACE.
• Metal chelation reduces burn-induced lipid aldehydes and rises lipid aldehyde dehydrogenases.

## 1 Introduction

Thermal injury is a dynamic progressive process that develops in the first few days after burn [[1], [2], [3], [4], [5], [6]] even without obvious clinical complications such as wound infection. The initial burn-induced inflammatory response, ischemia, and/or oxidative damage deteriorate the skin pathology, leading to the progression of partial-thickness second-degree burns to a deep partial-thickness burn or a deep second-degree burn becoming a third-degree burn [[5], [6], [7], [8]]. The progression from a deep partial-thickness burn to third-degree burn is a significant problem since second-degree burn wounds have the opportunity for re-epithelialization from the hair follicles near the bottom of the dermis. However, a third-degree burn wound cannot recover by itself and needs surgical grafting to reduce the risk of developing complications, such as infection, delayed wound healing, scar contracture, and subsequent aesthetic and functional disability. Although burn wound progression has been investigated [3,[9], [10], [11], [12], [13]], the current clinical burn wound management therapies are mainly focused on preventing burn wound infection and dehydration while stabilizing the patient systemically. The current therapeutic approaches do not include approved therapies that might limit or prevent burn wound progression [14]. These facts warrant the need for new clinical interventions to attenuate burn wound conversion in the early hours post-injury.

Extensive efforts have investigated therapies that will reduce burn wound conversion by targeting several mechanisms implicated in this process. These mechanisms include micro-thrombosis [4,9,15,16], wound dehydration [17], and specifically oxidative stress-induced inflammation via R oxygen species (ROS) production and lipid peroxidation [18,19]. While some studies have established interventions aimed to modulate oxidative stress-induced burn wound progression [18,20,21], we have focused on a therapeutic intervention that will block the formation of free radicals. For this purpose, we used the Livionex Formulation (LF) lotion containing disodium EDTA (ethylenediaminetetraacetic acid) as a metal chelator and methyl sulfonyl methane (MSM) as a permeability enhancer [22]. Using the rat model of brass comb burn, we found LF lotion to limit burn progression while concomitantly decreasing the accumulation of reactive lipid aldehydes and protecting the aldehyde dehydrogenase isozymes [22]. Using the porcine burn wound model that mimics closely the human skin burn, we investigated whether LF lotion will limit burn wound progression by reducing oxidative damage and inflammation. Similar to our rodent studies [22], we created brass comb burn wounds (100 °C for 30 s) in young Yorkshire pigs, then applied undiluted LF lotion three times a day for three days and examined its effects on burn wound conversion at 72 h after the initial thermal injury. We found that the application of LF lotion onto the porcine brass comb burn wound protected from oxidative damage and inflammation and subsequent horizontal and vertical burn wound progression.

## 2 Materials and Methods

### 2.1 LF lotion and brass comb

LF lotion was provided by Livionex Inc. (Livionex, Los Gatos, CA) and consists of two generally regarded as safe (GRAS) components: EDTA disodium as the chelating agent and methyl sulfonyl methane (MSM) as a permeability enhancer [22]. The brass comb used in this study was a modified version of the Regas and Ehrlich model [16] used in our previous study [22]. This comb produces three 10 × 19 mm rectangles of burn sites separated by two 10 × 19 mm rectangles of unburned interspaces.

### 2.2 Animals

Four (4) Class A 3-month old (26–32 kg) female Yorkshire pigs were used in the current study. The animals were obtained from a local single breeding farm and individually housed with a 12-h light-dark cycle in a temperature- and humidity-controlled environment. The animals were allowed at least one week of acclimatization before any manipulation. The animals were given a standard porcine diet (LabDiet® 5084, PMI Nutrition, IN) and water *ad libitum*. The Institutional Animal Care and Use Committee of the University of Texas Medical Branch at Galveston approved all animal manipulations reported in this study. All animal experiments were conducted following the National Institute of Health and the ARRIVE guidelines. Housing and care of the pigs met the National Research Council guidelines and were ensured by trained veterinarians.

### 2.3 Experimental protocol

**Fig. 1:**
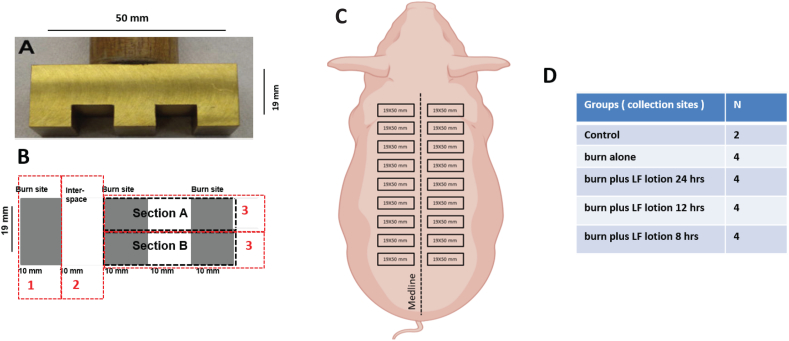
Study design. (A) Dimensions of the brass comb mold used for the burn procedure. (B) Tissue collection design as specified in the Methods section, (C) Demarcation of the burn sites as located on the dorsum of the Yorkshire pigs. Nine (19 × 50 mm) size burns were placed on each side of the dorsum 10 mm distal to the middle line resulting in 18 burn sites per animal. (D) Treatment group randomization for each burn site. N = burn sites.

Animals were premedicated with an intramuscular injection of ketamine and xylazine before inhalation anesthesia with 2–3% of isoflurane in room air via facemask. The pigs were then weighed, and the dorsum of each pig was completely clipped with an electrical clipper in a separate preparation room. The animals were then transferred to the operating room, placed on the operating table in a prone position, pre-oxygenated with 40% oxygen via inhalation mask, intubated and inhalation anesthesia maintained by 1–3% isoflurane for the whole experimental period. A venous line was established in an ear vein, and infusion of Ringer's lactate solution continued for the entire length of the experiment. As illustrated in the animal experiment design (Fig. 1), the animal's dorsum was marked on each side with two rows of 19 × 50 mm rectangles, resulting in nine 19 × 50 mm rectangles on each side of the dorsum and a total of 18 rectangles per animal. Each rectangle was about 20 mm apart from the other and about 10 mm distal to the middle line.

### 2.4 Randomization

Since the burn size is too small to induce a systematic effect (less than 2%TBSA burn), adjacent outlined rectangles on each animal were randomly divided into the following treatment groups: control without burn or lotion (n = 2 sites); burn alone (n = 4 sites); burn plus LF lotion one time per day (every 24 h, n = 4 sites); Burn plus LF lotion two times per day (every 12 h, n = 4 sites); Burn plus LF lotion three times per day (every 8 h, n = 4 sites). Our recent study using the rat brass comb model included a vehicle-only group that contains a lotion with MSM but no EDTA [23]. However, the vehicle-treated burn sites did not show any significant improvement of the burn-induced pathology. Therefore, we excluded the vehicle-treated group in subsequent studies.

### 2.5 Burn procedure

A 316 g brass comb was preheated in boiling water (100 °C) for 3 min (min) before use. The contact burn was induced by applying the preheated comb on the marked area for 30 s with no additional pressure. The burn-only sites received contact thermal injury with no additional treatment. The burn plus LF lotion rectangles received contact thermal injury followed by the application of LF lotion. For the burn plus LF lotion groups, the application of LF lotion started 15 min after the injury and was repeated every 8, 12, or 24 h each day for up to 72 h. No secondary dressing was used for these animals. The control sites did not receive any thermal injury. We only presented data from the 8- and 24-h’ time point in this manuscript.

After the first application, the wound area was cleaned every time by removing all debris and the dried layer of lotion was applied the previous time. LF lotion was applied to the whole burn wounds, including burn sites and unburned interspaces. After the burn procedure, animals were allowed to recover on the operating table. Once spontaneous breathing stabilized, they were extubated and transferred back to their cages. The animals had free access to water and a regular diet. For pre-and post-operative analgesia, transdermal fentanyl patches (100 mcg/hr) (Duragesic® 100, Janssen Pharmaceutics, NJ) were applied on the dorsal neck behind the ear 24 h before the procedure and kept in place using surgical staples plus a protective layer of adhesive transparent dressing. For additional analgesia, Buprenorphine (0.01–0.05 mg/kg IM) or Buprenorphine-SR (0.12–0.3 mg/kg SC) was given if needed.

### 2.6 Sample harvesting

All samples were harvested 72 h after-burn under anesthesia as described above for the burn procedure. Each wound was excised and then perpendicularly cut into three tissue blocks as illustrated in the study design Fig. 1: block 1: burn site only (10 × 19 mm); block 2, one interspace only (10 × 19 mm); block 3, two burn sites with one interspace in the middle (30 × 19 mm) that were divided into two sections A and B. The burn sites (blocks #1) and the interspaces (blocks #2) were cut into small tissue pieces that were frozen immediately for protein extraction. The tissue sections A and B of two burn sites one interspace (blocks #3) were fixed in 10% neutral buffered formalin for histology. Embedded tissues were cut into 5-μm sections with each containing three segments of skin tissues (two burn sites with an interspace between them), and the sections were stored at −20 °C until further processing for histology, immunohistochemistry, or TUNEL labeling.

### 2.7 Western blot analysis

As previously described [24], frozen skin tissues were homogenized in cold RIPA lysis buffer (25 mM Tris-HCl pH7.6/150 mM NaCl/1% NP-40/1% sodium deoxycholate/0.1% SDS) supplemented with 1% protein inhibitors cocktail, 10 mM β-Glycerophosphate (disodium salt), 4 mM Sodium pyrophosphate (decahydrate), 10 mM sodium fluoride, and 10 mM sodium orthovanadate (all from Sigma Aldrich, St Louis, MA). The protein concentration was determined using the bicinchoninic acid (BCA) assay. Twenty μg proteins were reduced, denatured, and separated in a 12% polyacrylamide gel. The proteins were then transferred onto polyvinylidene difluoride (PVDF) Plus membranes (Amersham Hybond-P, GE Healthcare, Buckinghamshire, UK). The membranes were then blocked and incubated overnight with primary antibodies (1:500 to 1:2000) then washed and incubated with horseradish peroxidase-conjugated secondary (1:1000) for 1 h at room temperature. The bound immune complexes were visualized using the enhanced chemiluminescence (ECL) Plus Western blotting detection system and Hyperfilm ECL (Amersham, Buckinghamshire, UK). The primary antibodies used in this study include rabbit anti-IL-6 (no. ab9324, Abcam), rabbit anti-TACE (no. 3976, Cell signaling, Danvers, MA), rabbit anti-ALDH1A1 (no. ab52492, Abcam, Cambridge, MA), and rabbit anti-TNF-a (no. 11948, Cell signaling, Danvers, MA). The blots were then stripped and immunoblotted for beta-actin or GAPDH (Santa Cruz Biotechnology, CA). Densitometry analysis was performed using the NIH Image J software (NIH). Data were normalized to the loading control.

### 2.8 Histochemistry and immunohistochemistry (IHC–P) of paraffin-embedded sections

For histochemistry (IHC), and as previously described [22], skin sections were de-paraffinized, rehydrated, and stained per the manufacturer instructions with Hematoxylin-Eosin (Hematoxylin Stain Harris Formulation, cat. #SL90-16 and Eosin Y, Cat. #SL98-16, StatLab, McKinney, TX), Masson's trichrome (Cat. #HT15-1 KT, Sigma-Aldrich Corporation, St. Louis, MO), or hematoxylin phloxine saffron (HPS) stain (Cat. #k023, Poly Scientific R&D Corp., Bay Shore, NY).

For IHC, skin sections were de-paraffinized, rehydrated, and treated with citrate buffer for antigen retrieval. Sections were then washed with 0.01 *M* PBS, quenched for 10 min in 3% hydrogen peroxide/methanol, washed with PBS, and blocked for 1 h in a blocking solution (5% normal goat serum/2% BSA/0.1% cold fish skin gelatin/0.1% Triton X-100/0.05%Tween 20/0.05% sodium azide in 0.01 *M* PBS). Tissue sections were then washed, blocked with avidin/biotin, and incubated overnight at 4^o^C with the primary antibody for mouse monoclonal anti-vimentin (V9) (#347-1, Sigma-Aldrich, St. Louis, MO). The next day, sections were washed with PBS, and incubated for 90 min with secondary biotinylated antibodies, washed, and processed in the dark for 1 h with ABC reagents (Standard Vectastain ABC Elite Kit; Vector Laboratories, Burlingame, CA). The sections were then developed with a mixture of DAB substrate (brown) (Dako, Carpinteria, CA) followed by counterstaining with 0.5% methyl green. Primary antibody titration and specificity were carried out at the beginning of the IHC experiment. The primary antibody step was omitted for negative controls.

### 2.9 Terminal deoxynucleotidyl transferase biotin-*d*-UTP nick-end labeling (TUNEL)

TUNEL labeling was used to label the 3′-hydroxyl termini in the double-strand DNA breaks generated during apoptosis. Frozen formalin-fixed paraffin-embedded skin sections were de-paraffinized, rehydrated, and then processed for TUNEL staining as described previously [22,25] with modification. The sections were incubated in 0.85% NaCl and 0.01 M PBS for 5 min, fixed in 4% paraformaldehyde in PBS for 15 min and washed with PBS. The sections were then incubated with proteinase K (20 μg/ml) for 10 min, washed with PBS, and fixed in 4% paraformaldehyde for another 5 min. After PBS wash, the sections were blocked with avidin/biotin solution (Invitrogen, Carlsbad, CA) for 15 min each, incubated in TUNEL reaction buffer (30 mM Tris-HCl, pH 7.2, 140 mM sodium cacodylate, and 1 mM CoCl_2_) for 10 min at room temperature, and then incubated in a humidified chamber for 90 min at 37 °C with TUNEL reaction mixture containing biotin-16-dUTP (10 nmol/ml) (Roche, Basel, Switzerland) with 200 U/ml terminal transferase (TdT). TdT was omitted for negative controls. Sections were then washed, incubated with ABC reagents in the dark for 60 min before staining with DAB substrate and counterstaining with 0.5% methyl green.

We used an Olympus BX53 digital microscope equipped with Cell Sense software to visualize staining and acquire all the images.

### 2.10 Microscopic scoring and measurement

Vertical injury progression in the burn sites and the effect of LF lotion on vertical burn progression were demarcated by scoring the microscopic depth of cell death including epithelial and endothelial cell death and follicle shafts labeled with TUNEL staining. Burn progression was also assessed by measuring the depth of vessel blockade labeled by HPS staining, and the depth of mesenchymal cells death labeled by vimentin. Vimentin is a type III intermediate filament protein expressed in mesenchymal cells, endothelial cells, macrophages, melanocytes, and lymphocytes. Vimentin expression is activated in early apoptotic cancer cells to signal cell survival pathways during DNA damage [26]. HPS staining differentiates between the most common connective tissues collagen (stained yellow), muscle, and cytoplasm (stained pink). The aforementioned morphological changes were viewed under (4×, or 10×, or 20× objectives) to ensure the pathological changes and measured in micrometers (μm) under a lower power objective (2×) using the CellSense Program. Quantification was performed in the middle of the burn sites in each sample as shown in the figures. Scoring was carried by two operators with one of them blinded to the treatment. Each score was recorded when agreed on by the two operators.

The depth of necrotic epithelial and endothelial cells was defined as the deepest vertical location of TUNEL-stained epithelial cells in the epidermis, hair follicles, and sebaceous glands. The depth of TUNEL-labeled endothelial cells lining the interior lumen of blood vessels of various diameters was also scored. This includes small arteries, arterioles, capillaries, venules, small veins, and veins. The depth of cell death was scored as elucidated in Table 1.

**Table 1:** Microscopic scoring of cell death.

Score	Depth of cell death
0	no lesion
1	lesions limited to the epidermis
2	lesions extended down to the upper half of the dermis
3	lesions extended down to the lower half of the dermis
4	lesions extended down to the upper half of the hypodermis
5	lesions extended down to the lower half of the hypodermis
6	lesions extended down to skeleton muscles

The depth of blood vessel blockade was defined as the lowest vertical location from the epidermal basement showing dilated blood vessels filled with denatured pink clots in HPS stained sections. The depth of mesenchymal cell death was demarcated by negative vimentin immunostaining at the lowest vertical location from the epidermal basement of any skin structural elements, such as blood vessels and cells including fibroblasts, macrophages, leukocytes, etc.

### 2.11 Statistical analysis

Normally distributed data were compared by standard parametric tests (GraphPad Prism, San Diego, CA) Data were presented as the mean ± SD. One-way ANOVA in conjunction with Tukey's post hoc test was used to stratify and determine differences among groups. Differences were considered significant at p < 0.05.

## 3 Results

### 3.1 Burn size

The total body surface area (TBSA) of the pigs used in the current study was 6669 ± 316 cm^2^, calculated according to Kelley's formula (TBSA in cm^2^ = 734 (body weight in kg) [27]. Depending on the individual animal's body weight and the number of comb wounds induced, the burn sizes were varying from 0.68% to 1.57% resulting in an average burn size of 1.19 ± 0.39%.

### 3.2 LF lotion reduces oxidative stress and inflammation and horizontal burn wound progression

**Fig. 2:**
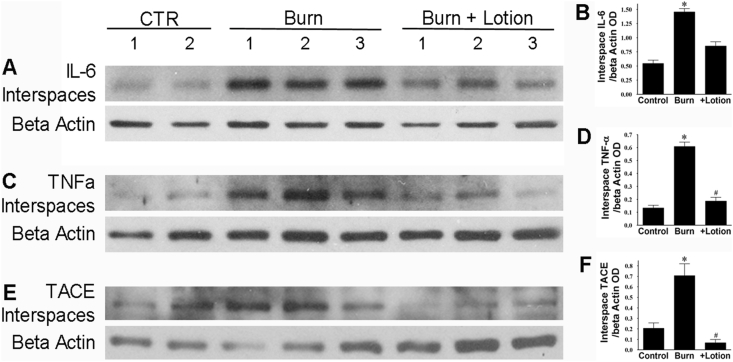
LF lotion reduces burn-induced inflammation in the interspaces. Western blotting expression of interspace IL-6, TNFα, and TACE. *Left*, representative interspace IL-6 (A), TNFα (C), and TACE (E) Western blots of control (CTR) burn alone and burn plus undiluted LF lotion application started 15 min right after the injury and repeated every 8 h thereafter, respectively. *Right*, OD ratio of interspace IL-6 (B) or TNFα (D) or TACE (F) vs. beta-actin. Control non-burned (n = 4), burn untreated (n = 6), and burn plus lotion (n = 6) sites were quantified for each marker. *, p < 0.05 vs. Control; ^#^p < 0.05 vs. burn alone.

**Fig. 3:**
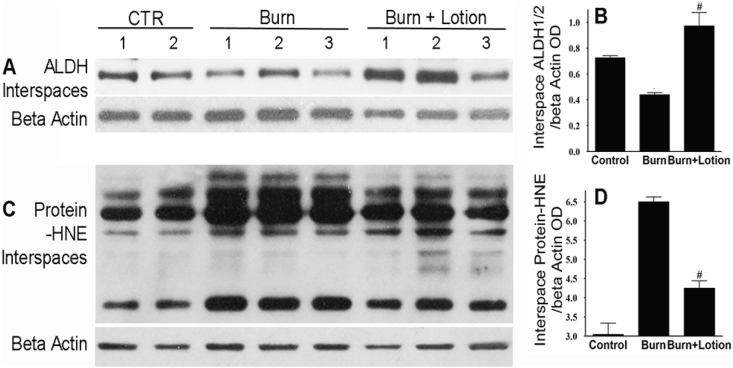
LF lotion rescues the burn-induced decrease in Aldehyde dehydrogenase protein expression. Western blotting expression of interspaces ALDH1/2 and protein-HNE. *Left*, representative interspace ALDH1/2 (*A*) and protein-HNE (*C*) Western blots of control (CTR), burn alone and burn plus undiluted LF lotion application started 15 min right after the injury and repeated every 8 h thereafter. *Right*, OD ratio of interspace ALDH1/2 (*B*) or protein-HNE (*D*) vs. beta-actin. Control non-burned (n = 4), burn untreated (n = 6), and burn plus lotion (n = 6) sites were quantified for each marker *, p < 0.05 vs. Control; ^#^p < 0.05 vs. burn alone.

Since the unburned interspaces, in the burn-untreated sites, did not bear a direct contact thermal exposure, any inflammatory response or morphological changes in the interspaces after burn will be the result of a horizontal burn progression. As shown in Fig. 2, Fig. 3, a standard 100^o^C- brass comb burn for the 30s induces a marked increase in the protein expression of inflammatory markers and lipid aldehyde production in the unburned interspaces. Horizontal burn wound progression increased the protein expression of IL-6, TNF-α, TNFα Converting Enzyme (TACE), and protein HNE to 2.7-fold, 4.7-fold, 3.4-fold, and 2.1-fold of their corresponding unburned controls levels, respectively.

Application of LF lotion every 8 h, during the first 72 h after injury, significantly reduced the inflammatory response and lipid aldehyde production. The protein expression of IL-6, TNF-α, TACE, and protein-HNE were reduced to 59%, 31%, 10%, and 65% of their corresponding burn alone values (all p < 0.05), respectively (Fig. 2, Fig. 3). Additionally, the burn significantly reduced ALDH1expression in the unburned interspaces to 40% compared to unburned normal control (p < 0.05) (Fig. 3A and B). The application of the undiluted LF lotion completely rescued the burn-induced decrease in ALDH1 protein expression (p < 0.05).

Morphologically, and as shown in section 3.4, the treated interspaces showed similar expression of vimentin immunoreactivity, TUNEL labeling, or HPS staining to the levels seen in the controls with no mesenchymal, epithelial or endothelial cell death and no blood vessel blockage in all layers (Fig. 6, Fig. 7, Fig. 8). These data histologically demonstrate that the application of LF lotion attenuates horizontal burn wound progression.

**Fig. 4:**
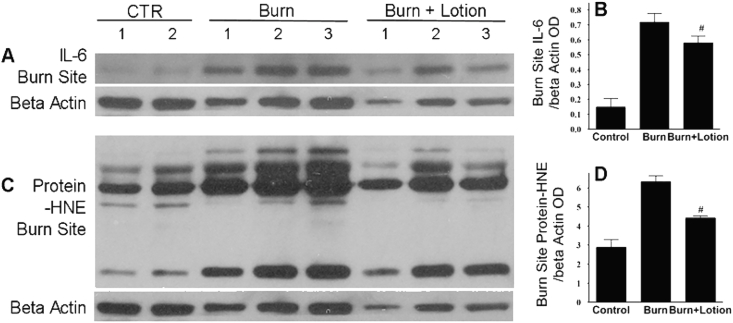
LF lotion reduces inflammation in the burn sites. Western blotting expression of burn sites IL-6 and protein-HNE.: Left, representative burn site IL-6 (A) and protein-HNE (C) Western blots of control (CTR), burn alone and burn plus undiluted LF lotion application started 15 min after the injury and repeated every 8 h thereafter. Right, optical density (OD) ratio of burn site IL-6 (B) or protein-HNE (D) vs. beta-actin. Control non-burned (n = 4), burn untreated (n = 6), and burn plus lotion (n = 6) sites were quantified for each marker *p < 0.05 vs. corresponding controls; ^#^p < 0.001 vs. burn alone.

**Fig. 5:**
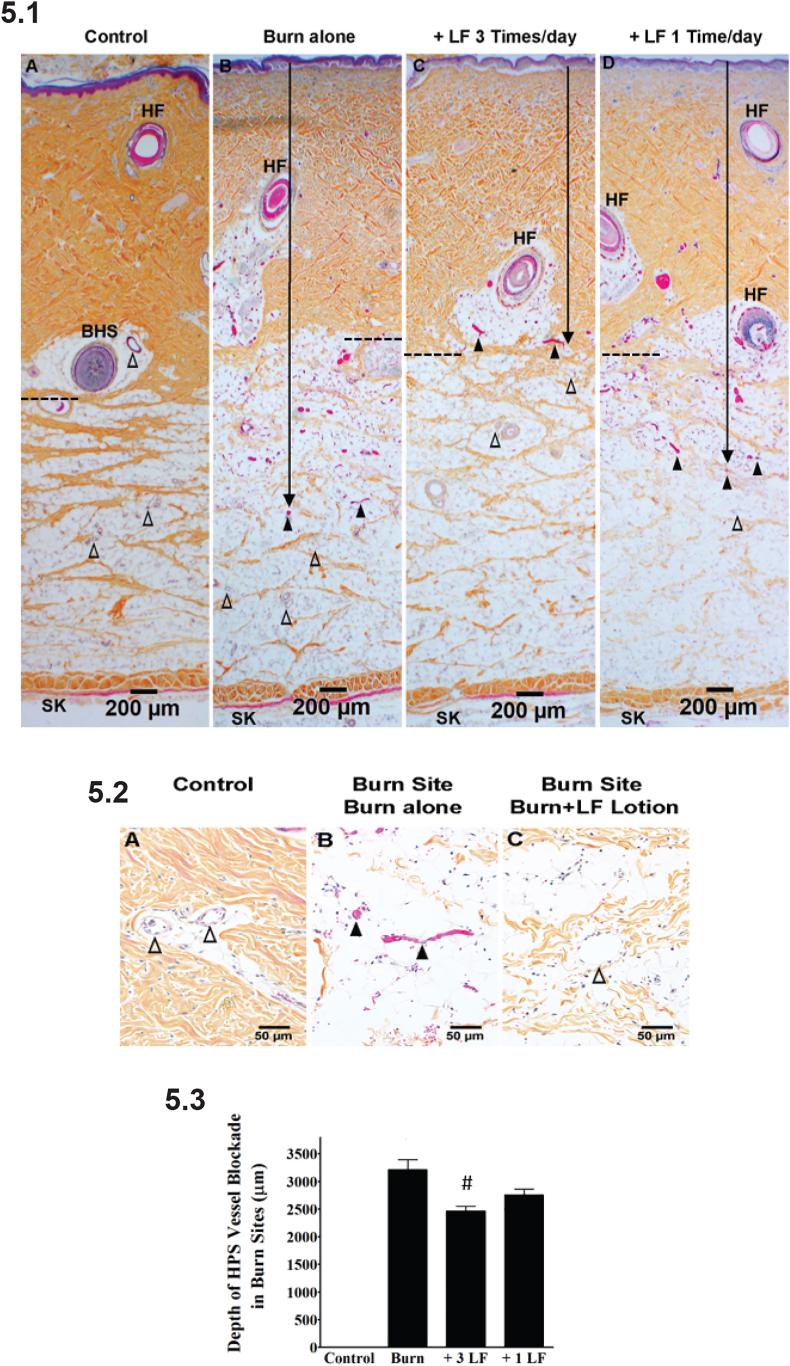
LF Lotion reduces vertical burn wound progression. 5.1. Representative microphotographs of HPS staining showing the depth of vessel blockade in burn sites of the porcine brass comb burn model. The black arrow marks the depth of the vessel blockade. The black dashed boxes in Fig B to C mark the deepest location of endothelial cell death in each experimental condition, respectively. 5.2. Higher power of the boxes in Fig. 5.1A, 5.1 B, and 5.1 C, respectively. 5.3. Quantification of HPS depth in the burn sites. (A) control without burn or lotion; (B) 72 h after a 30-sec burn alone; (C) 72 h after a 30-sec burn plus topical LF lotion application every 8 h started 15 min post-injury. (D) 72 h after a 30-sec burn plus topical LF lotion application every 24 h started 15 min post-injury. The black dashed lines mark the border of the dermis and hypodermis. The dashed boxes in panel 5.1 are magnified in panel 5.2. SK, skeletal muscle; BHS, the base of the hair shaft; HF, hair follicle; △, patent small blood vessels; ▲, blocked vessels filled with denatured clots. Scale bar = 200 μm (5.1), 50 μm (5.2). *p < 0.05 vs. Control. ^#^p < 0.05 vs. burn alone.

**Fig. 6:**
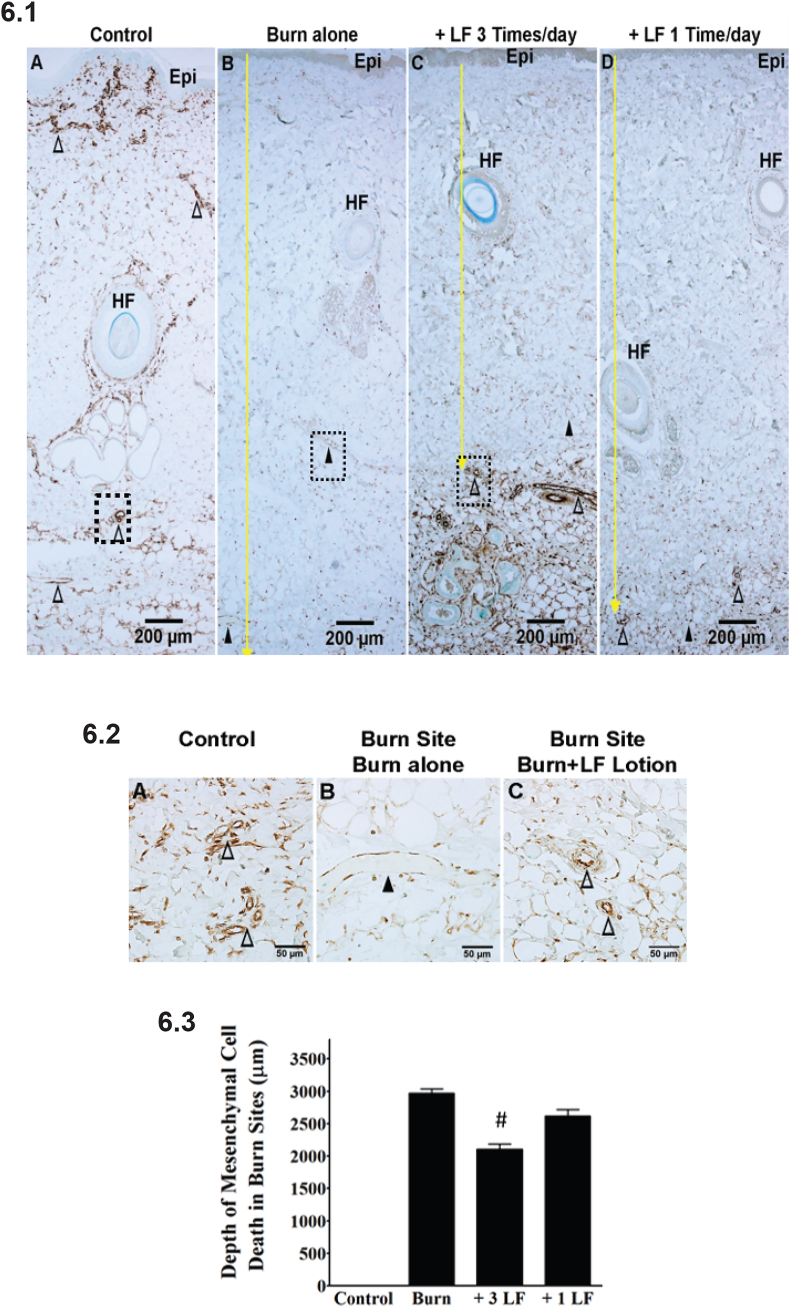
Mesenchymal cell death is reduced by LF lotion. 6.1. Representative microphotographs of vimentin staining showing the depth of mesenchymal cell death in burn sites of the porcine brass comb burn model. 6.2. Vimentin staining in high power magnification of the dashed boxes in Fig. 6.1A, B, and 6.1C, respectively. 6.3. Quantification of vimentin staining showing the depth of mesenchymal cell death in burn sites of the porcine brass comb burn model. (A) control without burn or lotion; (B) 72 h after a 30-sec burn alone; (C) 72 h after a 30-sec burn plus topical LF lotion application every 8 h started 15 min post-injury. (D) 72 h after a 30-sec burn plus topical LF lotion application every 24 h started 15 min post-injury. The yellow arrow marks the depth of cell death. The dashed boxes in panel 6.1 are magnified in panel 6.2. Epi, epidermis; HF, hair follicle; △, positively stained patent small blood vessels; ▲, negatively stained vessels filled with or without denatured clots. Scale bar = 200 μm (6.1), 50 μm (6.2).*p < 0.05vs. Control; ^#^p < 0.05 vs. burn alone. (For interpretation of the references to color in this figure legend, the reader is referred to the Web version of this article.)

**Fig. 7:**
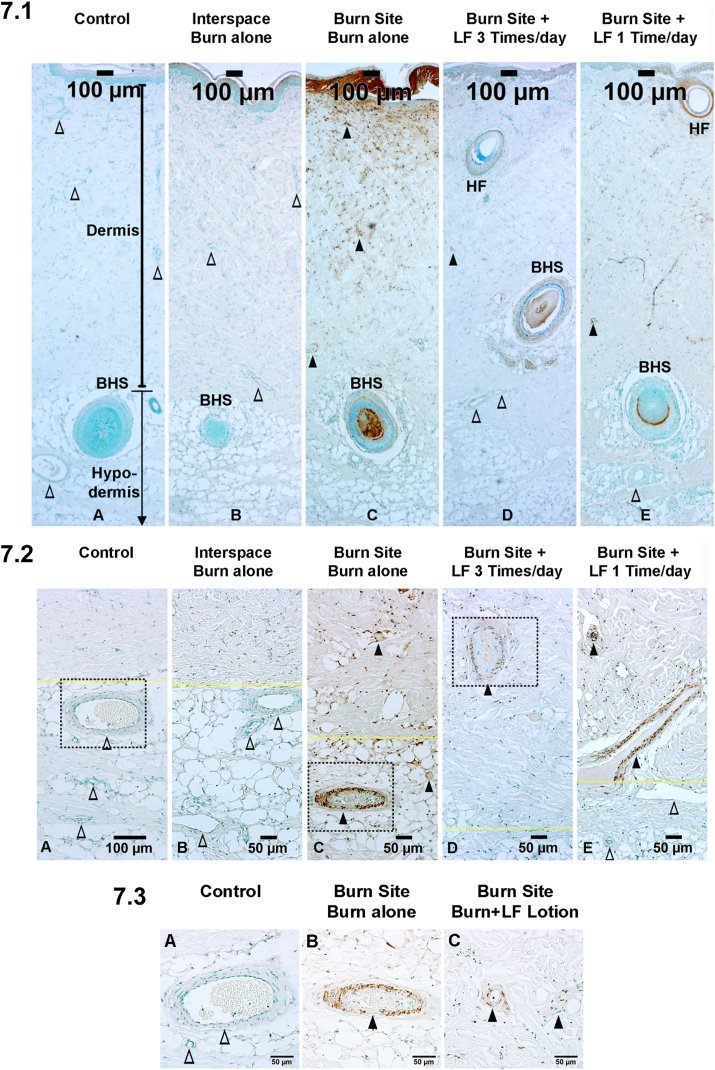
LF lotion attenuates epithelial and endothelial cell death. 7.1. Representative microphotographs of TUNEL labeling showing the depth of epithelial cell death of the porcine brass comb burn model. The black line with ends on both sides marks the depth of the dermis. The black line with the end on the top and arrow on the bottom marks the depth of hypodermis. 7.2. Representative microphotographs of TUNEL labeling showing the depth of endothelial cell death of the porcine brass comb burn model. The yellow lines mark the border of the dermis and hypodermis. 7.3. Higher power of the boxes in Fig. 7.2A, C, and 7.2D, respectively. X (A) control without burn or lotion; (B) interspace, 72 h after a 30-sec burn alone; (C) burn site, 72 h after a 30-sec burn alone; (D) burn site, 72 h after a 30-sec burn plus topical LF lotion application every 8 h started 15 min post-injury. (E) burn site, 72 h after a 30-sec burn plus topical LF lotion application every 24 h started 15 min post-injury. BHS, the base of the hair shaft; HF, hair follicle; △, patent blood vessels without positive TUNEL staining; ▲, positive TUNEL staining of endothelial cells of the vessels filled with or without denatured clots. Scale bar = 100 μm (7.1), 50 μm (7.2), and (7.3). (For interpretation of the references to color in this figure legend, the reader is referred to the Web version of this article.)

**Fig. 8:**
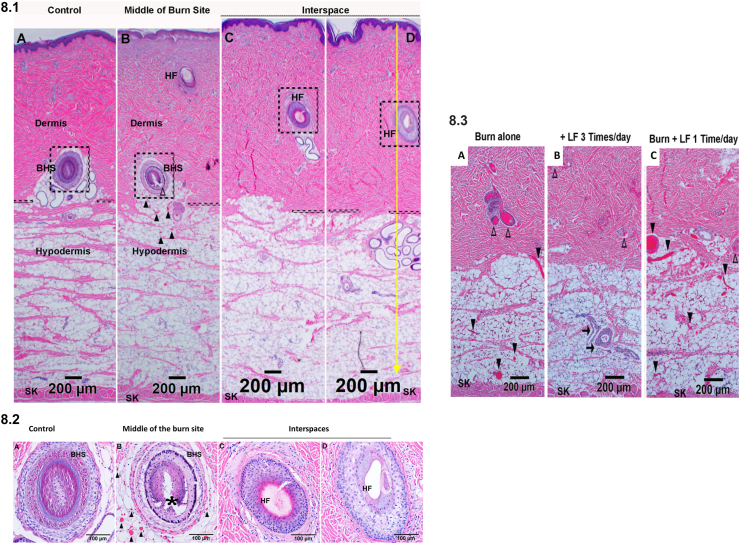
LF lotion preserves skin morphology and reduces both vertical and horizontal burn wound conversion. Representative microphotographs of H&E staining showing 8.1. Vertical and horizontal thermal injury progression from burn sites and interspaces of burn-only sections. 8.2. High power magnification of the dashed boxes around the hair follicles in Fig. 8.1 A, 8.1 B, 8.1 C, and 8.1 D, respectively. The black dashed lines mark the border of the dermis and hypodermis. (A) Control; (B) middle of burn site, 72 h after a 30-sec burn alone; (C) middle of one half of the interspace; (D) the middle of one whole interspace (the yellow arrow line marks the middle line of a whole interspace). 8.3. LF lotion applications 3 times per day (B) did prevent vessel blockade from progressing down to sub-dermal areas while one application a day (C) application did not. (A), 72 h after a 30-sec burn alone, (B) 72 h after a 30-sec burn plus topical LF lotion application every 8 h started 15 min post-injury, (C) 72 h after a 30-sec burn plus topical LF lotion application every 24 h started 15 min post-injury. HF, hair follicle. SK, skeletal muscle. BHS, the base of the hair shaft. △, hair follicle epithelial cell death and tissue destruction in the upper part of the hypodermis of burn site; ▲, blocked vessels filled with denatured clots. Scale bar = 200 μm (8.1 and 8.3), 100 μm (8.2). (For interpretation of the references to color in this figure legend, the reader is referred to the Web version of this article.)

### 3.3 LF lotion decreases vertical burn wound progression

The effects of LF lotion on vertical burn wound progression were assessed by immunoblotting and by scoring the vertical progression of cell death.

Burn induced a 4.8-fold increase in the protein expression of IL-6 in the burn sites (p < 0.05) that was reversed by 19% when treated by LF lotion (p < 0.01) (Fig. 4A and B). Similarly, burn-induced a 2.2-fold increase in HNE protein expression in the burn sites (p < 0.05) that was reduced by 30% when treated with LF lotion compared to the burn alone group (p < 0.05) (Fig. 4C and D). These results indicate that LF lotion reduced the inflammatory response and active aldehyde production in the burn site as a whole.

Quantitative image analysis of histological HPS staining (Table 2) shows 1) the microscopic measurements of the depth of blood vessel blockage (HSP staining) and mesenchymal cell death (vimentin staining) in mm and 2) scoring of the depth of epithelial and endothelial cell death (TUNNEL staining) following the score scale established in Table 1. Burn-induced blood vessel blockage reached 3.2 ± 0.5 mm into the middle of the hypodermis (Fig. 5.1B and 5.3). The average depth of vessel blockage in the LF lotion-treated burn sites was significantly reduced (p < 0.01) to approximately 2.5 ± 0.2 mm at the margin between dermis and hypodermis when the animals were treated 3 times a day (Fig. 5.1C and 5.3). The average depth of vessel blockage was reduced to 2.8 ± 0.3 mm when the animals were treated one time a day with the LF lotion (Fig. 5.1D and 5.3), which was less deep compared to burn alone but did not reach statistical significance (Fig. 5.1D and 5.3, Table 2).

**Table 2:** Microscopic measurements and scores of burn site pathology of interest in the porcine brass comb burn model.

Measurements and scoring	Control (n = 4)	Burn alone (n = 8)	Burn + 3 LF Lotion (n = 8)	Burn + 1 LF Lotion (n = 8)
Depth of vessel blockade *(mm)*	0 ± 0	3.2 ± 0.5*	2.5 ± 0.2*^#^	2.8 ± 0.3*
Depth of mesenchymal cell death*(mm)*	0 ± 0	3.0 ± 0.2*	2.1 ± 0.2*^#^	2.6 ± 0.3*
Depth of epithelial cell death *(score)*^*a*^	0 ± 0	3.8 ± 0.4*	2.6 ± 0.1*^#^	3.3 ± 0.4*
Depth of endothelial cell death*(score)*^*a*^	0 ± 0	3.9 ± 0.4*	2.4 ± 0.1*^#^	3.4 ± 0.6*

Notes: ^*a*^*,* Scoring of the microscopic depth of epithelial and endothelial cell death (TUNEL Staining) as detailed in Table 1. Data are presented as the mean ± SD. *, p < 0.05 vs. Control; ^#^p < 0.05 vs. burn alone.

Using the absence of positive vimentin immunoreactivity as an indicator of mesenchymal cell death, the average depth for mesenchymal cell death was around 3.0 ± 0.2 mm (middle of hypodermis) in the burn non treated sites (Fig. 6.1B and 6.3). The depth of mesenchymal cell death rose to the rim of the dermis and hypodermis when the burn sites were treated with LF lotion 3 times per day. This resulted in a depth of 2.1 ± 0.2 mm (p < 0.001, compared to burned untreated burn sites) (Fig. 6.1B and 6.3, Table 2). The depth of mesenchymal cell death reached 2.6 ± 0.1 mm (Fig. 6.1B and 6.3) when the burn sites were treated one time a day with LF lotion. These results demonstrated that the application of LF lotion every 8 h is more effective in reducing vertical burn injury progression compared to one application per day as evidenced by the reduction of the depths of blood vessel blockage with denatured clots and mesenchymal cell death.

Using the scoring scale illustrated in Table 1, the average depth of epithelial cell death in the untreated burn sites, evidenced by positive TUNEL staining in the middle of the upper half of hypodermis, was scored as 3.8 ± 0.4 (Fig. 7.1B, Table 2). This score was significantly reduced to 2.6 ± 0.1 in the burn sites treated 3 times a day with LF lotion (p < 0.05) compared to untreated wounds (Fig. 7.1C, Table 2). The average score of the depth of epithelial cell death in the burn sites treated 1 time a day with LF lotion was 3.3 ± 0.1 at the level underneath the dermis (Fig. 7, Table 2), which is less deep but not statistically different from the untreated burn wounds (p > 0.05).

Similarly, the average depth of TUNEL positive-endothelial cells was scored as 3.9 ± 0.4 (Table 2, using the score scale in Table 1) in the untreated burn sites (Fig. 7.2B and 7.3B) but significantly less deep (2.4 ± 0.1, p < 0.001) in the burn sites treated 3 times per day with LF lotion (Fig. 7.2C and 7.3C). In the burn sites treated 1 time a day with LF lotion, the average score for the depth of TUNEL positive endothelial cells was 3.3 ± 0.1 (Fig. 7.1E, Table 2), which is also less deep but not significant compared to the untreated burn wounds (p > 0.05).

### 3.4 LF lotion preserves skin morphology and reduces both vertical and horizontal burn wound conversion

HPS staining of the control non-burned sites shows no collagen discoloration, nor blood vessel blockage in the middle of interspaces (Fig. 5.1A). The majority of burn sites, however, exhibited lower HPS staining intensity in the upper portions of the dermal layers (Fig. 5.1B) when compared to control non-burn sites (Fig. 5.1A). A few burn untreated sites exhibited collagen discoloration in the upper portion of the dermis as previously reported after burn [28]. Blood vessel blockade with denatured blood clots was observed all over the dermis and the upper half of hypodermis in all burn untreated sites (Fig. 5.1B and 5.2B), but such pathology was not observed in the control non burned sections (Fig. 5.1A and 5.2A) or the non-burned interspaces.

Quantification of HPS vessel blockade shows that the application of LF lotion 3 times a day was more effective in reducing the burn-induced vessel blockade when compared to one application per day (Fig. 5.1D and 5.3, p < 0.05).

Analysis of H&E stained sections (Fig. 8.1B and 8.2B) showed that all burn-untreated sites exhibited universal and widespread necrosis with nuclear pyknosis of epithelial cells in the epidermis, hair follicle, and sebaceous glands. Necrosis was also noted in mesenchymal cells from the epidermis and upper dermis down to the lower dermis and even in the upper hypodermis (Fig. 8.1B and 8.2B). These data indicate vertical burn wound progression in our model.

Higher magnification of the burned untreated sites showed destruction of the hair follicles, nuclear pyknosis of all epithelial cells, and vessel blockade around the hair follicle (Fig. 8.2B). The hair follicle in control non-burned sites exhibited normal histology (Fig. 8.2A). In the burn-untreated sites, the middle of the burn untreated interspaces exhibited normal swine skin histology with no obvious morphological damage (Fig. 8.1D and 8.2D). However, the hair follicles in the interspace portion that is close to the burn sites (~2 mm from the demarcation of burn sites, Fig. 8.1C and 8.2C) showed necrotic epithelial cell death featuring nuclear pyknosis and suggesting horizontal burn progression from nearby burn sites.

### 3.5 LF lotion reduces mesenchymal cell death

Vimentin immunostaining in the untreated burn sites showed widespread mesenchymal cell death in the whole dermis and the upper half of the hypodermis (Fig. 6.1B and 6.2B), but not in the lower half of the hypodermis. LF lotion is more effective in reducing the depth of mesenchymal cell death when applied 3 times a day (Fig. 6D and E, p < 0.01) compared to one application per day.

TUNEL-labeling in the burn untreated sites showed epithelial cell death that includes the hair follicle shafts (Fig. 7.1B) and blood vessel endothelial cells (Fig. 7.2B and 7.3B) from the dermis down to the upper half of the hypodermis. Application of LF lotion reduces both epithelial and endothelial cell death assessed by TUNEL labeling (Fig. 7.1C, 7.2C and 7.3C, and Table 2).

Overall, our histological analysis demonstrates horizontal burn progression from nearby burn sites and vertical burn wound progression in the untreated burn sites and suggests that this burn model results in a full-thickness burn of the epidermis, dermis, and the superficial portion of the hypodermis. This model is characterized by the death of epithelial, endothelial, and mesenchymal cells from the epidermis, dermis, and the upper half of hypodermis, damage to hair follicle shafts involved in the skin regenerative potential, and blockage of blood vessels. This model is also suitable for the investigation of therapeutic interventions to reduce burn wound progression and improve wound healing.

## 4 Discussion

Following the burn, there is a surge in ROS and nitrogen species production [29], which is harmful and is implicated in inflammation, systemic inflammatory response syndrome, immunosuppression, infection and sepsis, tissue damage, and multiple organ failure [[30], [31], [32]]. Severe burn induces systemic inflammatory reactions by producing ROS and subsequent lipid peroxidation in many tissues, such as skin, plasma, liver, heart, and lung [21,[33], [34], [35], [36]]. Early studies have suggested that cellular oxidative stress is a critical step in burn-mediated injury and that antioxidant therapies aiming to inhibit ROS production or scavenge free radicals may reduce burn wound progression [35]. In addition, antioxidant therapies were shown to reduce burn wound infection, and accelerate wound healing [20,21,[37], [38], [39]] and reviewed in Ref. [40]. Deuterium labeled α-tocopherol administration also reduces markers of oxidative stress in the sheep model of burn and smoke inhalation injury [41].

While physiological metal levels are important for normal wound healing, metal insufficiency may impair normal wound healing. The levels of these metals are chronologically regulated to synchronize different processes involved in the regulation of the phases of wound healing. Copper, zinc, and selenium were also to increase after burn injury in a rat model of 10% TBSA 6 h after injury, the early time point examined in that study [42]. Burn injury induces depletion of calcium from the ER and increased cytosolic calcium levels [43]. Accumulation of extracellular calcium in the cytoplasm causes calcium influx into mitochondria to accelerate and disrupt normal metabolism leading to cell death. Calcium signaling pathways interact with other cellular signaling systems such as reactive oxygen species (ROS). The mutual interplay between calcium and ROS signaling systems has important implications for fine-tuning cellular signaling networks. However, dysfunction in either of the systems might affect the other system thus potentiating harmful effects, which might contribute to the pathogenesis of various disorders. While we did not measure the metals levels in this study, we have shown previously that burn increases ROS generation, lipid aldehydes and 4- Hydroxynonenal (HNE), and inflammatory cytokines, and these effects were attenuated by the metal chelator-containing LF lotion [22,23].

Our previous studies have shown that metal chelators reduce the deleterious effects of ROS generation in various disease models. We have shown that topical application of EDTA-containing eye drops exhibit protective effects against oxidative and inflammatory responses in rat models of glaucoma [44] and diabetic cataract [45]. Using the rat model of brass comb burn, we found topical application of EDTA-containing LF lotion in combination with a permeability enhancer methylsulfonylmethane (MSM) to reduce both horizontal and vertical burn wound progression [22]. The decreased burn wound conversion was demonstrated by a reduction in epithelial and endothelial cell necrosis, collagen denaturation, vessel blockade, and skeletal muscle damage in the burn sites. Similarly, the LF lotion-treated interspaces showed decreased skeletal muscle denaturation and discoloration, decreased vascular dilation and congestion in the capillary loops and subpapillary plexus, reduced inflammatory cells around dilated and congested vessels, and increased microscopic length of survived interspaces in the epidermis. LF lotion may be reducing oxidative damage by regulating the number of cells that produce extracellular reactive oxygen and nitrogen species [46]. The immune cells involved in the regulation of the host-defense response to injury like the polymorphonuclear neutrophils (PMNs) generate ROS that induces endothelial dysfunction by oxidation of cellular signaling proteins such as tyrosine phosphatases [46].

The present study used the same burn protocol (100^o^C for 30 s) and the same brass com model that we used for the rats [22], A similar model has been used previously to evaluate burn injury progression [13,47]. Here, we generated brass comb burn wounds in young Yorkshire pigs and followed with the application of LF lotion immediately after injury and every 8 or 24 h for 3 days. The EDTA-containing LF lotion protected against horizontal burn wound progression as shown by the significant reduction in the expression of inflammatory markers (IL-6, TNF-α, TACE) and lipid aldehyde production (protein-HNE) in the unburned interspaces when compared to their corresponding burn sites. Indeed, LF lotion rescued the burn-induced loss of ALDH1 protein expression in the interspaces.

Burn induces both local and systemic increases in inflammatory cytokines. Among those, IL-6 is known to activate the Th1 pro-inflammatory profiles and recruit other immune cell populations to the burn wound (reviewed in El Ayadi et al., 2020). IL-6 signaling was shown to reduce NO bioavailability and increased NADPH oxidase-derived superoxide, a combination that promotes oxidative stress [48]. TNFα is also increased in the wound microenvironment and topical treatments with anti-TNFα were shown to reduce the levels of downstream inflammatory mediators and attenuate secondary necrotic tissue expansion [49]. Besides, TNFα increases ROS generation in various models [50]. We found burn to increase both IL-6 and TNFα, and TNFα converting enzyme (TACE) in the burn site and interspaces of the rat and swine model of burn, an effect that was attenuated by immediate application of EDTA containing LF lotion. These data support the role of inflammation and pursuing oxidative damage in both vertical and horizontal burn wound progression.

Lipid peroxidation results from the oxidative degradation of cell membrane or subcellular organelle membrane phospholipid and polyunsaturated fatty acid (PUFA) and subsequent generation of highly damaging carbonyls such as aldehydes, ketones, and alkanes [51]. Reactive aldehydes permeate the cell membranes and intensify the effects of ROS. The damage caused by reactive aldehydes has been involved in many acute and chronic human diseases such as neurodegenerative diseases, alcoholic liver disease, diabetes, cancer, and various cardiovascular diseases [52,53]. 4-hydroxy-nominal (4HNE) and malondialdehyde (MDA) are highly reactive lipid peroxidation aldehydes that we found increased in both the rat [22] and swine brass comb model of burn suggesting excessive oxidative damage early after-burn that was attenuated by LF lotion. We also found burn to reduce the expression of Aldehyde dehydrogenases (ALDH1), a critical enzyme in aldehydes detoxification [54,55] suggesting reduced cell defense abilities after-burn that was restored by LF lotion in the interspaces. These data support the role of lipid peroxidation in horizontal burn wound progression. Burn wound progression is the culminating result of oxidative damage, cytokine release, apoptosis leading to cell death, collagen degradation, and tissue necrosis. LF lotion prevents burn wound progression by reducing ROS generation and subsequent lipid peroxidation to produce HNE and MDA. LF lotion also reduces the protein expression of Inflammatory cytokines IL-6 and TNF-alpha and subsequent inflammation and ROS generation.

More interestingly, the EDTA-containing LF lotion unveiled a significant protective effect on vertical burn wound progression by reducing the depth of blood vessel blockage with denatured clots, mesenchymal cell death as evidenced by the absence of positive vimentin immunostaining, and the depth of epithelial and endothelial cell death as demonstrated by positive TUNEL staining scores. This was further confirmed by a reduction in the protein expression of inflammatory markers (IL-6 and protein-HNE) in the LF lotion-treated burn sites compared to the untreated ones. These results suggest that the EDTA-containing LF lotion applied every 8 h significantly reduced the inflammatory response in the burn wounds and efficiently limited horizontal and vertical burn wound progression in this newly established porcine brass comb burn model.

Despite a large body of research on burn wound conversion, the current treatment guidelines for burn wounds do not contain approved therapies to limit or prevent burn wound progression [14] warranting further investigations. Further studies to explore the mechanisms underlying burn wound progression depend on the availability of reproducible and reliable animal models with appropriate burn severity and the availability, feasibility, and accuracy of the tools to monitor burn progression. Our rat brass comb burn model was modified from the original Regas and Ehrlich model by doubling the interspace (from 5 mm to 10 mm) [16]. The original Regas and Enlirch brass comb model with 5 mm interspaces was validated in both the rat and the pig model [47,56,57]. Burn wound progression in this model was characterized by measuring apoptosis, necrosis, and blood flow [13,56,57]. The modified model showed severe damage to all layers of the rat skin, the subcutaneous layer, skeletal muscles, and even the tissues beneath the skeletal muscle [22,23]. In the present study, the same burn model and settings produced severe damage to the porcine skin that can develop into a full-thickness injury if not treated promptly. The majority of the untreated burn sites showed indistinguishable dermis with necrosis but attached epidermis. Few burn sites exhibited indistinguishable dermis, where the different structures are not easily identified, with necrotic and detached epidermis. Quantitative analysis and scoring of the depth of mesenchymal cell death (absence of vimentin staining), blood vessel blockage (positive HPS staining), and epithelial and endothelial cell death (positive TUNEL staining) all confirmed that burn-induced damage reached the upper half of the hypodermis in the untreated burn sites, suggestive of a full-thickness third-degree burn 72 h after the initial injury. Application of LF lotion significantly reduced injury progression to the upper half of the hypodermis and limited the damage in the dermis.

The LF lotion-induced improvement was limited to the layer where the bases of the hair follicles are anchored and rescued the root of hair follicles suggesting that LF lotion may promote stem cell proliferation and differentiation to heal the wounds. This model is suitable for the investigation of molecular and cellular mechanisms underlying burn injury progression and to develop therapeutic interventions to limit burn wound progression and promotes wound healing.

As stated above, the LF lotion-induced improvement in the present porcine brass comb burn model is statistically significant but far from the ideal goal of full prevention of burn wound progression. This limited effect may be related to the time when the application of LF-lotion was started and the frequency of application. In the present study, LF lotion application started 15 min after the initial injury, a time that can be mimicked by real-time scenarios in emergency situations. We tested three application times, 8, 12, and 24 h, and reported data for the 8 and 24 h’ time points. We found that frequent applications every 8 h are more beneficial compared to one application per day. However, more frequent applications may increase the local concentration of the lotion that could reach the wound site.

Another possible reason for the moderate effect of LF-lotion in this model is that the formation of free radicals may not be the only mechanism underlying burn injury progression. Increased TNF-α production after burn, for example, maybe responsible for epithelial and/or endothelial cell death suggesting that therapy aiming to reduce TNFα production or activity may help. The initial burn and the subsequent burn wound progression may impair hair follicle stem cell proliferation and differentiation. Therapies aiming to rescue or stimulate hair follicle stem cell function may also be worth trying. Finally, since blood vessel blockage is a universal phenomenon, therapies aiming to prevent or limit the formation of vessel blockage are also appropriate to reduce burn wound progression.

## Declaration of competing interest

The authors declare no conflict of interest.
